# RE: Is There a More Practical Way for Screening Minimal Hepatic Encephalopathy?

**DOI:** 10.5152/tjg.2023.231972

**Published:** 2023-06-01

**Authors:** Deniz Eyice, Ayşe Merve Ok, Abdullah Sonsuz, Mustafa Şükrü Şenocak, Meliha Nur Durak, Sebati Özdemir, Billur Canbakan, Murat Tuncer, Ibrahim Hatemi

**Affiliations:** 1 Department of Internal Medicine, Cerrahpaşa School of Medicine, İstanbul University-Cerrahpaşa, İstanbul, Turkey; 2 Division of Gastroenterology, Department of Internal Medicine, Cerrahpaşa School of Medicine, İstanbul University-Cerrahpaşa, İstanbul, Turkey; 3 Department of Biostatistics, Cerrahpaşa School of Medicine, İstanbul University-Cerrahpaşa, İstanbul, Turkey

Dear Editor,

We read the letter^1^ with great interest, thank you for your comments. 

As the authors mentioned in their letter^1^ a smartphone-based application can be very useful for daily practice. We think that the main problem in these applications is the difficulty related to language. For example, encephalapp stroop test is in English and you can not change the language of the application. We think also grasping a pen is not the same as using a smartphone screen. For some patients, it is very difficult to use a small phone screen. 

We appreciate the authors’ comment and we thank them again for their interest in our article.^2^

## Declaration of Interests:

The authors have no conflict of interest to declare.
